# A roll of the dice: pathogen–host interaction and the evolution of disease susceptibility

**DOI:** 10.1017/S0031182025101492

**Published:** 2026-03

**Authors:** Paul Capewell, Philipp Olias, Brian Shiels

**Affiliations:** 1 School of Molecular Biosciences, College of Medical, Veterinary and Life Sciences, University of Glasgow, Glasgow, UK; 2 Institute of Animal Pathology, Vetsuisse Faculty, University of Bern, Bern, Switzerland; 3 Institute of Veterinary Pathology, Justus Liebig University, Giessen, Germany; 4 School of Biodiversity, One Health and Veterinary Medicine, College of Medical, Veterinary and Life Sciences, University of Glasgow, Glasgow, UK

**Keywords:** DNA-binding proteins, epigenesis, evolution, gene expression regulation, host–pathogen interactions, *Theileria annulata*

## Abstract

The probability that a disease will manifest is highly variable. Susceptibility to disease is influenced by genetic background, environment and lifestyle choices. In this review, we put forward the premise that evolution of disease susceptibility may be partially influenced by the interaction of divergent pathogen DNA-binding proteins with variable binding sites in the host genome. The hypothesis put forward is derived from recent data obtained from work on the protozoan parasite, *Theileria annulata*, together with related evidence from viral and bacterial pathogens that have been postulated to modulate host epigenome architecture. The pathogen proteins highlighted have the potential to mimic functions of mammalian epigenome organisers linked to a range of disease syndromes. It is feasible, therefore, that the evolutionary relationship between pathogen and host impacts susceptibility to a range of conditions, such as autoimmune disorders and cancer, which are not directly linked to pathogen infection.

## Graphical abstract

**Figure:**
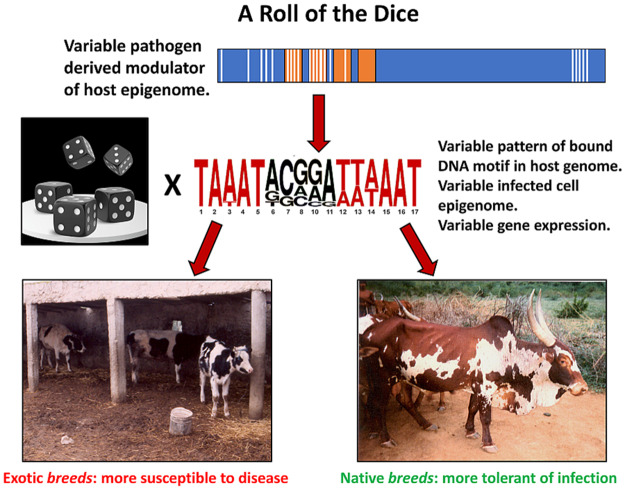

## Introduction

Disease manifestation varies widely between individuals, even when infected with the same pathogen. Pathologies can be linked to specific genetic mutations where the occurrence of disease is virtually guaranteed. The outcome for many conditions, however, is more unpredictable. Moreover, whether a disease occurs or not can be influenced by external factors, such as environment and lifestyle, which appear to operate independent of genetic information inherited by the affected individual. Understanding how these factors promote disease in some individuals but not others is key to the prediction of disease risk and promotion of health in a population.

The adaptation of hosts and pathogens to display mild disease or asymptomatic infection is theorised to occur via a co-evolutionary arms race that promotes an equilibrium between host survivability and pathogen transmission, reducing pathology and increasing tolerance to infection (Glass et al., 2012; Sironi et al., 2015). In contrast, novel infection of hosts that have not co-evolved with a pathogen would be expected to be more susceptible to severe disease (Glass et al., 2012; Seal et al., 2021). This has been shown for numerous zoonotic infections and by the transfer of high-yield, exotic livestock breeds into endemic regions of disease (Glass, 2001; Seal et al., 2021). Despite co-evolution of pathogen–host interaction towards milder disease outcomes, there is still the unpredictable potential for members of a tolerant population to suffer acute disease and for those of susceptible breeds to show limited clinical signs (Glass et al., 2005).

To address how variability in disease susceptibility can be generated, our work has aimed to identify factors that promote the markedly different outcomes that occur when different cattle breeds are infected with the tick-borne parasite *Theileria annulata*, the causative agent of tropical theileriosis. Data indicate that susceptibility to this disease is, in part, mediated by direct interactions between pathogen-derived factors and the host genome that influence chromatin structure and gene expression. The importance of the epigenetic landscape is often overlooked when seeking to identify susceptibility-linked heritable traits but could make a significant contribution given the known role of the epigenome in numerous infectious and non-infectious diseases (Davis et al., 2021).

## Pathogenesis and cellular transformation in tropical theileriosis

Tropical theileriosis is a disease of economic importance to livestock production and is endemic over much of Asia and North Africa (Minjauw and McLeod, 2003). Acute disease results in dysregulation of the immune response and lung pathology that mirrors numerous viral diseases. Both large and small production systems are susceptible, and the impact on poorer subsistence farmers is catastrophic. European cattle breeds (*Bos taurus*), such as Holstein, show a greater susceptibility to acute disease than breeds native to endemic regions, like Sahiwal (*Bos indicus*) (Glass et al., 2012). This should be expected, as native breeds that have co-evolved with *Theileria* would be closer to reaching an equilibrium in the trade-off between host survivability and pathogen transmissibility. Efforts have been made, including the national programme in India (Singh, 2015), to improve productivity by crossbreeding tolerant native cattle breeds with European breeds. While this has mitigated losses, infected crossbred animals still show clinical signs and lower production than European breeds (Larcombe et al., 2019).

Previous studies have confirmed that Sahiwal cattle are tolerant to *T. annulata* infection when compared to Holstein cattle (Glass et al., 2005). A pro-inflammatory response (cytokine storm) has been observed in infected Holstein calves, allowing postulation that acute disease pathology is linked to parasite-mediated dysregulation of the host immune response (Glass et al., 2012). In support of that premise, transcriptome analysis on *T. annulata-*infected leukocytes, *B. taurus* (Holstein) vs *B. indicus* (Sahiwal), has found that tolerance is associated with altered expression of hundreds of infection-associated bovine genes that operate in innate immunity and oncogenesis, demonstrating a multifaceted phenotype (Jensen et al., 2008; Larcombe et al., 2022). However, despite showing a statistically significant breed associated trend across both sets of samples, many genes showed a degree of variable transcript level within each breed. Given the complicated nature of the data, it is not clear how differential susceptibility to tropical theileriosis arises. One model for this is that divergent interaction between pathogen factors and host chromatin promote different epigenetic landscapes that lead to a wide range of phenotypes, both between and within breed: from tolerance to acute disease.

The major point of host–pathogen interaction associated with pathology in tropical theileriosis is the macroschizont-infected leukocyte. Establishment of the infected cell is accompanied by transformation into a cancer-like cell that metastasises to several organs, including the lungs. Destruction of the lymphoid system and massive pulmonary oedema then occur (Irvin and Morrison, 1987), and differences in breed susceptibility have been linked to these events (Chaussepied et al., 2010). Transformation of leukocytes occurs via constitutive activation of bovine transcription factors, such as NF-κB and AP1, to promote infected cell survival and oncogenesis (Shiels et al., 2006), but these factors also act as pro-inflammatory mediators. Activation of these host cell ‘Janus-like’ transcription factors is modulated by parasite-dependent reorganisation of the infected cell gene expression network (Durrani et al., 2012). Thus, while some NF-κB target genes show elevated expression, others are negatively regulated, and expression of interferon-stimulated genes is moderated (Oura et al., 2006; Durrani et al., 2012). More recent work extends these findings and indicates that differential modulation of the infection-associated transcription network is linked to breed susceptibility (Larcombe et al., 2022). Thus, significant differences (<0.1 FDR) in the expression of hundreds of infection-associated genes were identified between macroschizont-infected cell lines derived from 6 infected Sahiwal calves compared to 5 Holsteins. Pathway analysis highlighted an enrichment of genes and pathways associated with innate immunity and cholesterol biosynthesis, and a modulated type I interferon response was prominent. These results demonstrate that the expression of genes with potential to impact the outcome of infection is actively manipulated by the parasite in a breed-dependent manner. Because most intracellular pathogens hijack the host cell machinery to construct a niche that promotes their survival, the information gained from the *Theileria* model may apply to other pathogen–host interactions.

A common pathogen hijacking strategy deployed to establish an intracellular niche is the export of molecules that co-opt regulation of host cell gene expression. Pathogens subvert regulation of gene expression by corrupting host transcription factor (TF) activity and by altering host chromatin structure to modulate TF accessibility (Cheeseman and Weitzman, 2015; Davis et al., 2021). *Theileria annulata* likely deploys both strategies. Direct manipulation of transcription factor state is demonstrated by constitutive activation of NF-ĸB via hijacking of the IĸB kinase signalosome (Heussler et al., 2002), while parasite factors encoded in *TashAT* gene cluster are indicated as modulators of host chromatin structure (Swan et al., 2001; Shiels et al., 2006; Larcombe et al., 2022). TashAT proteins travel to infected leukocyte nuclei, bind AT-rich DNA and contain AT-hook domains that are organised in a manner similar to mammalian high mobility group A (HMGA) proteins. HMGAs operate by binding and affecting various AT-rich genetic (DNA) motifs to modify chromatin structures and methylation, regulating transcription factor accessibility and gene expression. HMGAs therefore act as hubs of nuclear function (Reeves, 2001) and have a major role in development, stem cell maintenance, cholesterol biosynthesis and the inflammatory response (Treff et al., 2004; Henriksen et al., 2010; Vignali and Marracci, 2020). These pathways are also differentially modified by *Theileria* infection, depending on whether the infected cell line was derived from a tolerant or susceptible host (Larcombe et al., 2022). Due to the similarity between mammalian HMGAs and parasite TashAT proteins, we proposed that they act in a related manner and that the pathogen modifies host chromatin and TF accessibility by binding AT-rich genetic motifs to influence the epigenetic landscape and disease susceptibility. In support of this model, many loci ascribed as causal variants for disease susceptibility have been linked to regions of chromatin accessibility (Maurano et al., 2012).

## Breed-specific DNA motifs and selection of epigenetic architecture

**Figure 1.**
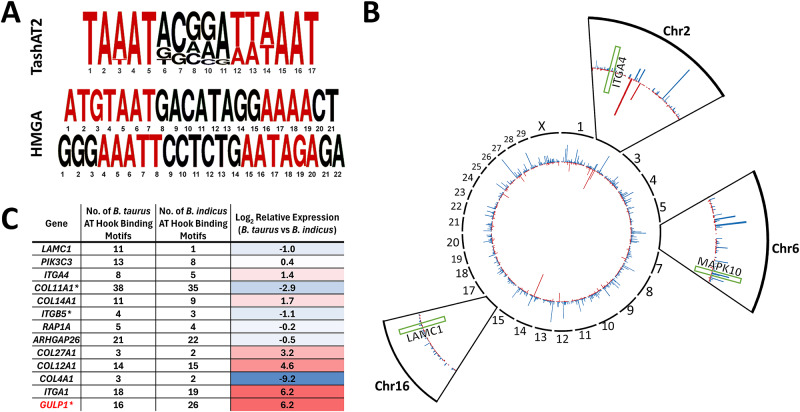
Differences in the number and location of AT-hook binding site motifs between *Bos taurus* and *Bos indicus* genomes (A) Consensus DNA sequence motif bound by parasite TashAT2 compared to mammalian HMGA. AT rich regions are in red. (B) CIRCOS summary plot of genes displaying a different number of TashAT2 binding sites in *Bos indicus* (red) and *Bos taurus* (blue). Zoomed views for chromosomes 2, 8 and 16: genes in the EGF and integrin signalling pathways with different numbers of TashAT2 binding site motifs and differing in expression between Holstein and Sahiwal infected cells are indicated by green boxes (ITGA4, MAPK10 and LAMC1). (C) Integrin pathway genes differentially expressed between *T. annulata* infected *Bos taurus* vs *Bos indicus* infected cell lines displaying different numbers of predicted TashAT2 binding site motifs (data derived from Larcombe et al., 2022). Relative expression is shaded using a red-to-blue gradient, with red indicating higher values and blue indicating lower values. GULP1 (highlighted red) has been experimentally validated as a modulated target gene in a TashAT2 transfected-BoMac cell line (Durrani et al., 2023). Figure 1 long description.

Expression of a TashAT encoding gene (*TashAT2*) in a transfected bovine macrophage cell line altered transcript levels of more than 800 bovine genes (Durrani et al., 2023). Pathway analysis indicated involvement in focal adhesion, transforming growth factor (TGF)-β, axon guidance and integrin signalling with similarity to pathways regulated by mammalian HMGA2 (focal adherence, TGF-β, integrin signalling) (Singh et al., 2015), supporting postulation that the parasite factor acts as an HMGA mimic. Moreover, 184 genes (*P* < 0.1) modulated by TashAT2 also display differential expression between Sahiwal and Holstein infected cells, with enrichment of genes operating in Integrin signalling (*P* = 0.04) and Axon guidance (*P* = 0.02). A further link was established by comparison of the published *B. taurus* and *B. indicus* genomes for AT-rich nucleotide motifs bound by TashAT2 (Larcombe et al., 2022). The results showed that the pattern (number and positions) of the TashAT2-bound consensus motif differs substantially between the 2 genomes (Figure 1) and that more than 45% of genes differentially expressed between Sahiwal and Holstein infected cells feature at least one putative TashAT2 binding motif (*P* < 0.001). Within this cohort, there is enrichment in pathways modulated by infection and differences in the number of TashAT2 binding motifs between genes of *B. indicus* vs *B. taurus*. An example is the EGF/PI3K pathway, linked to transformation of the infected leukocyte and modulated by mammalian AT-hook proteins (Wang et al., 2021). Furthermore, altered expression of integrin signalling genes is associated with virulence of the *Theileria* infected leukocyte (Haidar et al., 2015) and can occur via TashAT2 (Singh et al., 2015) and HMGA1 (Reeves et al., 2001). Based on these findings, it can be postulated that variability in the arrangement and, potentially sequence, of motifs bound by AT-hook proteins vary between breeds and possibly individual animals. Such variability could impact on epigenome architecture and the resulting expression level of multiple genes linked to disease susceptibility. How the variability in motif arrangement within the host genome is generated is not known. The nucleotide motif bound by HMGA AT-hook proteins and TashAT2 is only semi-conserved and consists of a pair of AT-rich sequences interspersed with a region higher in GC content (Swan et al., 2001; Su et al., 2020; Larcombe et al., 2022). It is probable that these motif patterns have a level of variability between breeds and possibly within breed populations. It is also possible that evolution of the TashAT2-bound motif pattern in the host genome could be influenced by selective pressure, as interaction with a lethal pathogen infection could remove, over time, individuals with patterns that give rise to fatal disease (Tseng et al., 2021). Mammalian HMGA proteins are known to operate in important developmental pathways and are linked to haematopoiesis, epithelial–mesenchymal transition and adipogenesis (Su et al., 2020; Vignali and Marracci, 2020). Thus, selective breeding for particular traits may influence the pattern of motifs (and epigenome architecture) encoded by genomes of cattle breeds that are differentially susceptible to pathogen infection.

## Genetic diversity and the evolution of pathogen–host interaction

**Figure 2.**
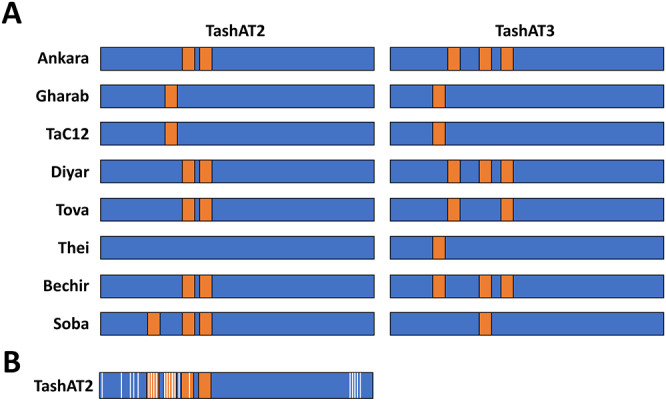
Amino acid diversity and rearrangement of AT-hook motifs in TashAT2/3 alleles (A) Pattern of AT-hook motifs predicted from allelic DNA sequences of TashAT2 and TashAT3. The regions containing the TashAT locus were extracted from 8 publicly available genomes (ENA project ID: PRJEB65114) previously aligned to the C9 Ankara reference strain using BEDtools. The number and positions of AT-hook motifs were identified using SMART and the core (GRP) motif for each putative site validated manually. Relative positions of each AT-hook domain are denoted Orange. (B) The TashAT2 sequences were compared using JCoDA to identify sites of positive selection of amino acid substitution. The relative positions of sites with a probability of (dN/dS > 1) greater than 0.9 (white) were overlaid on a composite cartoon of the TashAT2 alleles. Figure 2 long description.

An additional potential contributor to altered infected cell gene expression is diversity in the pattern of AT-hook domains across TashAT2/3 alleles. In the analysis of (Weir et al., 2010), Ten major arrangements were identified across 8 sequenced isolates. These findings have been validated, by analysis of parasite genomes derived from multiple clonal cell lines. The results identified TashAT2/3 alleles displaying variable numbers and spacing of AT-hooks. Moreover, dN/dS analysis provides evidence for positive selection of amino acid substitution primarily within the first 2 AT-hook domains of TashAT2 and towards the C-terminus of the protein (Figure 2). The negatively charged C-terminal tail of HMGA proteins show variable phosphorylation patterns that are thought to influence DNA binding efficiency or protein partner binding, which may contribute to cellular transformation (Sgarra et al., 2009; Su et al., 2020). In analogy, the substitutions highlighted for the C-terminal region of TashAT2 overlap with predicted PEST motifs (Shiels et al., 2006) and phosphorylation sites for protein kinases. Post-translational modification of PEST regions in nuclear proteins are considered important for partner binding and modulation of function, including oncogenic activity (Sarfraz et al., 2021).

The data for selection of variant pathogen AT-hook domains, together with evidence that TashAT alleles display geographical substructuring (Weir et al., 2010), implies that *T. annulata* has co-evolved with local host genetic backgrounds to recognise divergent DNA target motifs. In this scenario, the random probability of disease would be influenced by both the genetic background of the infecting parasite populations and the genome of the infected host, leading to a wide range of infection outcomes, between and within animal breeds. Over evolutionary time the pathogen–host relationship has produced *B. indicus* genomes that possess patterns of (AT-rich) DNA binding motifs that, in general, promote tolerance; while European, Bos taurine breeds, selected for productivity traits in the absence of pathogen pressure, display greater susceptibility. The model predicts that (1) parasite TashAT proteins contribute to the construction of chromatin architectures that promote gene expression changes linked to a spectrum of disease outcomes; (2) differences in the pattern of motifs that interact with AT-hook proteins are linked to breed morphological traits and disease susceptibility and (3) the binding of variant TashAT factors to variant host genome contributes to disease susceptibility.

## Analogous viral and bacterial epigenetic modulators

A relevant question in relation to the above model is whether it is applicable for other intracellular pathogen–host interactions. Evidence for multiple pathogen factors manipulating the host epigenome has been generated (Silmon De Monerri and Kim, 2014; Cheeseman and Weitzman, 2015; Davis et al., 2021). The 2 examples of pathogen nucleomodulins most related to *T. annulata* are the Epstein–Barr nuclear antigen 1 (EBNA1) of Epstein Barr Virus (EBV) and the ankaryin A (AnkA) protein of *Anaplasma phagocytophilum* (Table 1).

**Table 1.** Comparison of 3 protozoal, viral and bacterial nucleomodulins that act as (putative) analogues of host architectural transcription factors Table 1 long description.

Pathogen	Effector protein	Host target sequence	Putative analogue	Proposed mode of action	Amino acid diversity
*Theileria annulata*	TashAT2	AT-rich DNA motifrelated to HMGA binding site.	HMGA1/2	Binds to host DNA. Modulates chromatin structure; Integrin signalling, TGFβ/SMAD3, EGF/PIK3 pathways.	Yes: AT hooks, C-terminal PEST.
*Epstein-Barr virus*	EBNA1	AT-rich DNA flanking DBD binding sites.	HMGAs	Binds to host DNA. Modulates epigenetic protein kinase A pathways.	Yes: DBD domain.
*Anaplasma phagocytophilum*	AnkA	AT-rich DNA of matrix attachment regions.	SATB1	Binds to host DNA. Modulates chromatin organisation & epigenetic landscape. Repression of CYBB expression and other host cell defence genes.	Yes: correlation with strain tropism for host species.

Like *Theileria*, EBV infection is linked to a transformed host cell phenotype and associated pathology (Burkitt’s lymphoma, Hodgkin lymphoma). Latent virus infection requires attachment of the EBV episome to host chromosomes. This is achieved, in part, via the DNA binding functions of EBNA1 (Frappier, 2012). DNA binding has been shown to involve at least 3 domains. Two domains (CBD1 and CBD2) possess AT-hook motifs that show similarity to mammalian HMGA1a (Kanda et al., 2013) and *Theileria* TashAT2/3. Furthermore, the episomal tethering function of EBNA1 can be replicated by HMGA1. Genome-wide mapping of EBV episome attachment sites identified enrichment of AT-rich DNA, flanking C-terminal EBNA1 DNA binding domain (DBD) sites and the epigenetic H3K9me3 mark (Kim et al., 2020). These sites were associated with transcriptional repression of genes with neural functions or genes operating in the protein kinase A pathway. Thus, EBNA1 binds to host DNA and, like TashAT2, has been postulated to act as an analogue of HMGAs (Coppotelli et al., 2013) to modulate the host cell epigenetic landscape resulting in altered gene expression. In further analogy to TashAT2, study of EBNA1 gene sequences from different viral strains identified a high level of sequence divergence (Bell et al., 2008). While evidence for amino acid substitution in the AT-hook domains is limited, the majority (68%) of isolates predict alterations in the C-terminal DBD. It was proposed that these substitutions may be linked to the evolution and geographical origin of the viral isolate.

*Anaplasma phagocytophilum* is a tick-borne, Gram-negative bacteria placed in the order Rickettsiales. It is the cause of anaplasmosis in domestic animals and humans. Human granulocytic anaplasmosis is the third most common tick-borne disease in Europe and the USA. The outcome on infection is variable; most patients have mild clinical signs but for 36%, infection results in hospitalisation, with 7% of clinical cases requiring intensive care (Dumler, 2012). *Anaplasma phagocytophilum* infects neutrophils and, like *Theileria*, pathology is linked to a proinflammatory cytokine storm with similarity to COVID-19 (Ramanujam et al., 2021). In further analogy to *T. annulata*, infection of the host cell results in activation of PI3K/AKT and NF-ĸB pathways to protect against apoptosis (Sarkar et al., 2012). An extensive reprogramming of gene expression (Borjesson et al., 2005) that moderates production of proteins involved in innate immunity operates (Garcia-Garcia et al., 2009b).

Modulation of gene expression is generated through alteration of host cell chromatin architecture. Wide-scale manipulation of the epigenome is mediated by the AnkA protein that is secreted and translocated to the neutrophil nucleus via an importin-b-, RanGTP-dependent mechanism (Kim et al., 2022). AnkA manipulates the epigenome by binding to runs of AT-rich DNA and recruiting histone deacetylase 1 (HDAC1) to the *CYBB* promoter region. Recruitment of HDAC1 causes silencing of *CYBB* expression, promoting pathogen survival (Rennoll-Bankert et al., 2015). DNA binding is achieved by 4 central ankyrin repeats, together with a C-terminal AT-hook like domain with a core RGR motif. Chromatin immunoprecipitation (ChIP) studies indicate that AnkA acts genome wide to generate large scale changes to the epigenome of the infected cell (Garcia-Garcia et al., 2009a; Dumler et al., 2016). Moreover, AnkA binds to regions of the genome similar to those of matrix attachment regions and lamina associated domains. Therefore, AnkA could act as an analogue of host transcription factors, such as the special AT-rich binding-protein-1 (SATB1), also a target of HIV-1, trans-activator of transcription modulatory activity (Kumar et al., 2005), that organise chromatin architecture into topological domains within the nucleus, that in turn, influence the transcriptional programme of the cell (Sinclair et al., 2014).

Similar to TashAT2/3 and EBNA1, AnkA displays a high degree of sequence divergence across bacterial strains (Scharf et al., 2011). Most nucleotide changes are nonsynonymous, predicting amino acid substitution. While no geographical subclustering has been found, different AnkA gene clusters correlate with strain tropism for different host species, suggesting a level of co-evolution and adaptation. There is no evidence, to date, that diversity in AnkA impacts on disease outcome. However, functional diversity is thought possible (Scharf et al., 2011): and it may be noteworthy, that enriched binding of AnkA to DNA upstream of target gene transcriptional start sites displayed significant variability between the 3 different human genomes investigated (Dumler et al., 2016).

## Conclusions

**Figure 3.**
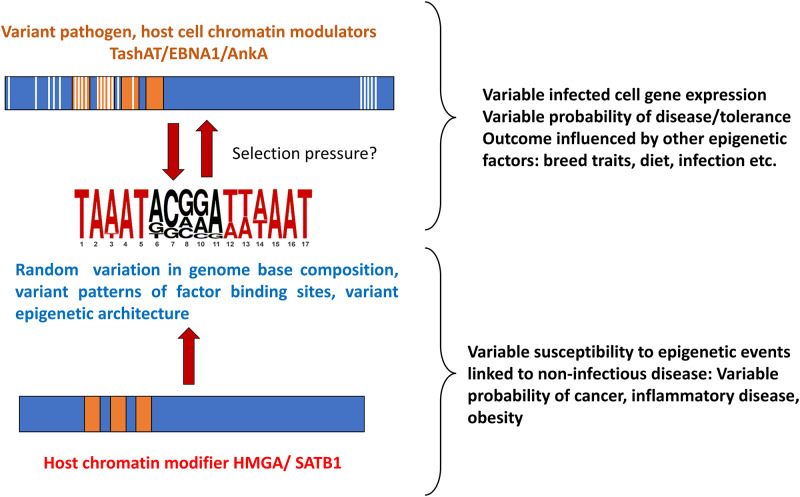
Overview of the proposed model for pathogen-host interaction and evolution of disease susceptibility. Figure 3 long description.

How genome diversity and environmental factors interact to influence susceptibility to disease is not fully understood. The model postulated (outlined in Figure 3) whereby variant DNA motifs interact with divergent regulatory proteins to construct variable epigenetic landscapes, suggests a link between genome, environment and lifestyle which could be important for understanding how the response to infection can vary so widely. For example, the infected cell epigenome and susceptibility to theileriosis may be influenced by host genome, parasite genome, livestock environment/nutrition and infection of the bovine host by other pathogens. Moreover, pathogen factors may mimic host molecules (e.g. HMGA, SATB1) (Coppotelli et al., 2013; Sinclair et al., 2014; Durrani et al., 2023) that function as base composition readers (Quante and Bird, 2016) and shape chromatin architecture during embryonic development (Goolam and Zernicka-Goetz, 2017; Panchal et al., 2020; Su et al., 2020; Vignali and Marracci, 2020). These host factors have been implicated in a range of conditions, including neoplasia and disease linked to immune dysfunction (Reeves, 2001; Treff et al., 2004; Henriksen et al., 2010; Frömberg et al., 2018; Panchal et al., 2020; Vignali and Marracci, 2020). Based on the premise that pathogen infection could influence selection of genomes with particular patterns of motifs to which the host factors bind: further study of pathogen-host interaction has potential to provide information on how variable susceptibility to diseases unrelated to infection has evolved, with the caveat that individual outcomes can still depend on just a roll of the dice.

## Limitations of the model and future perspectives

The model outlined above has limitations and requires additional data. To date, it is based on 2 reference genomes representing *B. taurus* and *B. indicus*. Analysis of genome sequence representing multiple breeds of both subspecies is required; with the proviso that a degree of diversity in the TashAT2 binding site motif pattern (number, position and potentially sequence) is predicted between different breeds and between individuals within a breed. It may also be of interest to identify the gene expression profile of infected leukocytes, and the binding site pattern in the genome, of water buffalo (*Bubalus bubalis*). This could provide information on whether evolution of tolerance to *T. annulata* displayed by native cattle breeds and *Bubalus bubalis* reflects related or divergent mechanisms.

While pathogen modulation of the host epigenome may be a relatively common strategy (Bierne and Pourpre, 2020; Silmon De Monerri and Kim, 2014; Cheeseman and Weitzman, 2015), it is unknown how applicable the above model is to intracellular pathogens, in general. Consensus AT-hook domains are prevalent in eukaryotes but thought to be rare in prokaryotes (Aravind, 1998). AT-hook proteins are encoded by herpes viruses and AT-hook domains have been reported as shared between viral, host and prokaryotic DNA binding proteins (Dreyfus et al., 2018). Multiple extended nucleic acid binding AT-hook motifs, and other protein domains with potential to act as base composition readers, have been described (Filarsky et al., 2015; Quante and Bird, 2016). The model, therefore, may be applicable to other intracellular pathogens. Even if limited to the 3 examples given above; over evolutionary time, a considerable proportion of the human and animal population will have been exposed to these pathogen–host interactions.

Additional data could be obtained by: the assay for transposase-accessible chromatin with high-throughput sequencing of *B. indicus* and *B. taurus* infected leukocytes (Buenrostro et al., 2015), to compare regions of chromatin accessibility; ChIP-sequencing (Johnson et al., 2007), to functionally identify genomic locations that TashAT2 protein binds; comparison of the gene expression profile modulated by distinct TashAT2/3 alleles (Durrani et al., 2023); investigation of whether TashAT2 binding site diversity is driven by selection (Sironi et al., 2015) or has evolved passively (McShea, 1994).

## Figure 1 Long description

The image consists of three sub-images labeled A, B and C. In A, the consensus DNA sequence motif bound by parasite TashAT2 is compared to mammalian HMGA, with AT-rich regions highlighted in red. The sequences for TashAT2 and HMGA are displayed, showing distinct patterns of AT-rich regions. In B, a CIRCOS summary plot illustrates genes with varying numbers of TashAT2 binding sites in two genomes, marked in red and blue. The plot includes zoomed views for chromosomes 2, 8 and 16, highlighting genes in the EGF and integrin signaling pathways with different numbers of TashAT2 binding site motifs. These genes, such as ITGA4, MAPK10 and LAMC1, are indicated by green boxes. In C, a table lists integrin pathway genes differentially expressed between infected cell lines, showing different numbers of predicted TashAT2 binding site motifs. The table includes columns for the number of AT-hook binding motifs in two genomes and log relative expression values, shaded from red to blue to indicate higher and lower values, respectively. GULP1 is highlighted in red, indicating it has been experimentally validated as a modulated target gene in a TashAT2 transfected cell line.

Navigate back to Figure 1.

## Figure 2 Long description

The figure displays horizontal bar representations for multiple sample groups arranged in rows, each labeled with a distinct name. Two main columns compare patterns between two conditions, with each row containing a long rectangular bar divided into colored segments. Most of each bar is filled with a dominant color, while shorter contrasting segments are interspersed along the length, indicating regions of variation or distinct features. The positions and frequency of these contrasting segments differ between samples and between the two conditions, suggesting variability in structure or composition. In the lower portion, an additional panel presents a single extended bar with more frequent alternating segments, highlighting a more complex or detailed pattern. The layout emphasizes comparison across samples, showing how segment distribution changes between groups and conditions, with consistent alignment enabling visual tracking of differences across rows.

Navigate back to Figure 2.

## Figure 3 Long description

A scientific schematic with labelled blocks, arrows and a right-side curly bracket grouping outcome statements. A long horizontal bar made of many narrow vertical segments appears near the top. A second long horizontal bar made of many narrow vertical segments appears near the bottom. Between the two bars is a large DNA motif shown as a single line of letters, with smaller letters beneath it and a row of numbers beneath the smaller letters. Several arrows point between the bars and the DNA motif, including a pair of opposing vertical arrows between the top bar and the DNA motif and a single upward arrow between the bottom bar and the DNA motif. Text in the image is English. Text near the top reads: Variant pathogen, host cell chromatin modulators Text below that reads: TashAT slash EBNA1 slash AnkA Text near the opposing arrows reads: Selection pressure question mark The large DNA motif reads: TAAATACGGAATTAAAT Smaller letters beneath the motif read: taaataCGGAATTaaat Numbers beneath the smaller letters read: 1 2 3 4 5 6 7 8 9 10 11 12 13 14 15 16 Text near the upward arrow reads: Random variation in genome base composition, variant patterns of factor binding sites, variant epigenetic architecture Text near the bottom reads: Host chromatin modifier HMGA slash SATB1 Text grouped by the curly bracket reads: Variable infected cell gene expression Variable probability of disease slash tolerance Outcome influenced by other epigenetic factors: breed traits, diet, infection etc. Additional text near the lower right reads: Variable susceptibility to epigenetic events linked to non-infectious disease: Variable probability of cancer, inflammatory disease, obesity.

Navigate back to Figure 3.

## Table 1 Long description

The table compares three pathogen effector proteins by pathogen, the AT-rich host DNA sequences they target, the host architectural transcription factor they are proposed to mimic, their proposed actions, and whether they show amino-acid diversity. All three effectors—TashAT2 (Theileria annulata), EBNA1 (Epstein–Barr virus), and AnkA (Anaplasma phagocytophilum)—bind AT-rich host DNA, suggesting a shared strategy of chromatin modulation. TashAT2 targets an AT-rich motif related to an HMGA binding site and is proposed to act like HMGA1/2, influencing chromatin structure and pathways including integrin signaling, TGFβ/SMAD3, and EGF/PIK3. EBNA1 binds AT-rich DNA flanking its DNA-binding domain sites, is proposed to mimic HMGAs, and is linked to modulation of epigenetic protein kinase A pathways. AnkA binds AT-rich matrix attachment regions, is proposed to mimic SATB1, and is associated with chromatin reorganization and repression of CYBB and other host defense genes. Each effector is marked as having amino-acid diversity, attributed to AT hooks and a C-terminal PEST region (TashAT2), a DNA-binding domain (EBNA1), or correlation with strain tropism for host species (AnkA). The “putative analogue” and “proposed mode of action” entries indicate hypotheses rather than confirmed mechanisms.

Navigate back to Table 1.
